# Successful treatment of a patient with refractory nephrotic syndrome with PCSK9 inhibitors: a case report

**DOI:** 10.1186/s12882-017-0644-0

**Published:** 2017-07-06

**Authors:** Yuki Awanami, Makoto Fukuda, Yasunori Nonaka, Tsuyoshi Takashima, Keiichiro Matsumoto, Masatora Yamasaki, Motoaki Miyazono, Yuji Ikeda

**Affiliations:** Department of Internal Medicine, Saga University Faculty of Medicine, 5-1-1 Nabeshima, Saga, Japan

**Keywords:** Proprotein convertase subtilisin/kexin type 9 (PCSK9), Evolocumab, Nephrotic syndrome, Case report

## Abstract

**Background:**

The proprotein convertase subtilisin/kexin type 9 (PCSK9) inhibitor evolocumab is a low-density lipoprotein (LDL)-lowering drug with a new mechanism, which is currently available in Japan. Here, for the first time, we report the successful use of the PCSK9 inhibitor in a patient with refractory nephrotic syndrome.

**Case presentation:**

A 61-year-old woman was diagnosed with minimal change-type nephrotic syndrome in October 2012. She received prednisolone (PSL) and cyclosporin A (CyA), but she experienced several cycles of relapse and remission and was hospitalized in May 2016 due to relapse. However, in spite of steroid pulse therapy and adrenocorticotropic hormone (ACTH) administration, her urinary protein level did not improve. We started her on evolocumab with the expectation of equivalent LDL-lowering effects as seen with LDL apheresis. After that, the LDL cholesterol level and UP/UC were concomitantly decreased, and the serum albumin was increased. This was maintained even when we reduced the PSL dose. This suggests that evolocumab clinically improves the nephrotic condition.

**Conclusion:**

No other report has described the use of evolocumab for nephrotic syndrome (NS) or its effect on similar nephrotic conditions. We believe that the findings presented here are unique and may be beneficial when treating similar cases.

## Background

The efficacy of low-density lipoprotein apheresis (LDLA) for refractory nephrotic syndrome (NS) has been described [1], but whether or not its efficacy is due to a decrease in LDL levels is unknown. LDL-lowering medications other than LDLA have also been used as adjuvant therapy for NS, but no report has stated a sufficient reduction of urinary protein [2]. Recently, the proprotein convertase subtilisin/kexin type 9 (PCSK9) inhibitor evolocumab, which is a therapeutic agent with a new mechanism for dyslipidemia, became available in Japan, and it has been shown to decrease LDL more effectively than other available agents [3]. We used evolocumab before performing LDLA for a patient with refractory NS who did not show sufficiently response to an increase in the dosage of steroids or immunosuppressive drugs. Here, we report our experience with a case that exhibited a significant decrease in urinary protein level with our regimen.

## Case presentation

A 61-year-old woman was referred to our hospital for the onset of edema and proteinuria in October 2012. She was hospitalized, and the laboratory results showed TP 4.7 g/dL, Alb 0.7 g/dL, TC 580 mg/dL, and urine protein/urine creatinine ratio (UP/UC) 21.95 g/gCr, indicating nephrotic syndrome. We diagnosed her with minimal change-type nephrotic syndrome, because the selectivity index (SI) of the proteinuria was high (SI 0.11) and there were no specific pathologic findings on renal biopsy. She was started on 40 mg oral prednisolone (PSL) daily as the initial treatment. She achieved complete remission once, so we reduced the PSL dose to 5 mg. However, 100 mg cyclosporin A (CyA) had to be additionally administered because she experienced recurrence after 6 months; subsequently, she experienced several cycles of relapse and remission. On May 2016, she experienced her sixth recurrence while receiving 10 mg PSL and 75 mg CyA. She was hospitalized because her urinary protein level had not improved even after the PSL dose was increased to 20 mg.

Her medical and family history were unremarkable. She did not drink or smoke. Her allergic history was only limited to drug reactions, which is suspected to be due to sulfamethoxazole/trimethoprim, alfacalcidol and famotidine. When she was hospitalized, she was administered prednisolone 20 mg once daily, atorvastatin calcium hydrate 10 mg once daily, sodium gualenate hydrate 1.5 g once daily, limaprost alfadex 5 μg thrice daily, CyA 75 mg once daily, and alendronate sodium hydrate 35 mg once weekly. Her height was 152.3 cm, and her body weight was 53.9 kg. Her vital signs were as follows: body temperature 36.0 °C, blood pressure 126/76 mmHg, pulse rate 104 times/min, regular, and SpO2 96% (room air). She had pitting edema in both legs. The laboratory data showed leukocytosis without a shift to the left (white blood cells 14,600/μl), hypoproteinemia (serum total protein 6.0 g/dl), hypoalbuminemia (serum albumin 2.4 g/dl), hyperlipidemia (total cholesterol 358 mg/dl), increased levels of hepatobiliary enzymes (AST 24 U/L, ALT 28 U/l, LDH 310 U/l, ALP 185 U/l and γGTP 78 U/l), and positive urinary protein (UP/UC 19.3 g/gCr). Chest X-ray showed a cardio-thoracic ratio of 54.3% and lack of pleural effusion.

### Clinical course after admission (Fig. 1)

We followed up after hospitalization with PSL 20 mg and CyA 75 mg for 9 days, and we increased the CyA dose to 150 mg (blood concentration 2 h after administration was 1010 ng/mL), because there was no improvement in her urinary protein level. Ten days after increasing the CyA dose to 150 mg, her urinary protein level was still within nephrotic range (UP/UC 9.97 g/gCr) and her hypoalbuminemia worsened (Alb 1.6 g/dl). Therefore, after 24 days of hospitalization, we performed steroid pulse therapy (mPSL 500 mg every 3 days) and started her on 40 mg PSL orally and 150 mg CyA daily as after care. Nevertheless, this failed to improve her urinary protein level (UP/UC 14.85 g/gCr) and serum albumin (Alb 1.2 g/dL). Therefore, after 32 days of hospitalization, we administered 1 mg adrenocorticotropic hormone (ACTH), which has been reported to be effective for refractory nephrotic syndrome [4].

**Fig. 1:**
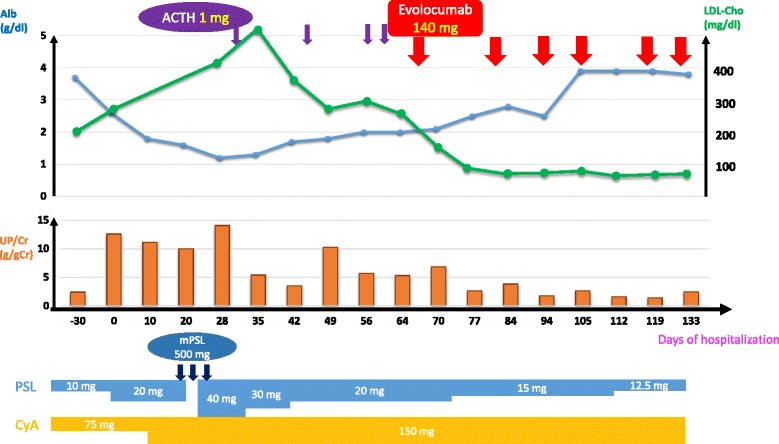
Clinical course

Subsequently, we reduced the PSL dose to 20 mg, as her UP/UC improved to 1.85 g/gCr and Alb increased to 2.0 g/dL. However, 1 week after that, her UP/UC worsened to 4.09 g/gCr, so we administered 1 mg ACTH for a total of 4 times. Nevertheless, hypoalbuminemia (Alb 2.0 g/dL) and nephrotic urinary protein excretion (UP/UC 8.83 g/gCr) sustained. We considered performing LDLA, as the lipid metabolism disorder persisted. However, we first decided to administer evolocumab because of its LDL-Cho-reducing effects, since performing LDLA increases the risk of extracorporeal circulation.

We administered 140 mg evolocumab in a single dose after 66 days of hospitalization. Two weeks later, the LDL-Cho level decreased from 199 to 43 mg/dl, the UP/UC decreased from 6.60 to 2.63 g/gCr, and the serum albumin increased from 2.0 to 2.5 g/dl. Assured of the effect of the treatment with evolocumab, we reduced the PSL dose to 15 mg while continuing evolocumab at a dose of 140 mg every 2 weeks.

Because the UP/UC was less than 2.0 g/gCr and was stable, the patient was discharged from the hospital. After her discharge, we reduced the PSL dose to 12.5 mg with the CyA dose of 150 mg, and the LDL-Cho and UP/UC subsequently dropped to 13 mg/dL and 1.41 g/gCr, respectively, and the TP and Alb increased to 6.2 g/dL and 3.9 g/dL, respectively.

As of May 2017, the patient had maintained incomplete remission (UP/UC 1.69 g/gCr, Alb 3.6 g/dL), so we could reduce the PSL dose to 6 mg with the CyA dose of 150 mg. Because her LDL-Cho decreased to 6 mg/dL, since February 2017, we changed the evolocumab dosage interval from every 2 weeks to once a month with an aim of maintaining the LDL-Cho level from 60 to 70 mg/dL.

## Discussion

In many cases, primary nephrotic syndrome can be controlled via steroid therapy with immunosuppressive drugs. However, there are some intractable cases, where various inventions are used to treat patients with little success [5]. We administered ACTH and considered re-biopsy anticipating potential focal segmental glomerular sclerosis (FSGS) because of resistance to therapy. However, we did not perform the latter procedure after considering the risk of complication caused by older age and long-term steroid use.

Dyslipidemia is a main symptom of NS. It promotes arteriosclerotic changes in the kidney and increases the risk of degeneration of renal function. A meta-analysis of patients with renal and cardiovascular diseases showed that statins decrease proteinuria and prevent renal function degeneration [2]. However, none of the studied have shown that statins contribute to improve proteinuria in nephrotic patients.

It has been shown that LDLA improves urinary protein levels in patients with refractory nephrotic syndrome, particularly patients with FSGS [1]. However, in this case, we were concerned about the potential complication of extracorporeal circulation due to the prolonged use of steroids. Therefore, we decided to administer evolocumab, a therapeutic agent with a new mechanism targeting lipid metabolism, because we expected it to strongly decrease LDL-C and improve urinary protein levels similar to that with LDLA.

Evolocumab is an anti-PCSK9 monoclonal antibody. PCSK9 is a serine protease that is produced and released into the circulation by the liver. It plays an important role in controlling LDL metabolism. LDL receptors on hepatocyte surface bind to LDL in the blood and take it into the hepatocytes. After LDL resolution, the receptors are recycled back to the hepatocyte surface. PCSK9 binds to the LDL receptor and inhibits this recycling, thereby decreasing LDL receptor expression on the hepatocyte surface. Evolocumab inhibits the binding of PCSK9 to the LDL receptor by selectively binding to PCSK9 and increasing LDL receptor expression on the hepatocyte surface. As a result, the blood LDL cholesterol level decreases. Kyubok Jin reported that PCSK9 levels were higher in patients with nephrotic syndrome than in controls and that the elevated PCSK9 levels were associated with an increase in LDL cholesterol and TG, based on the examination of blood PCSK9 levels in those patients [6]. Therefore, PCSK9 inhibitors may be able to resolve lipid abnormalities in patients with nephrotic syndrome.

No report has described the use of evolocumab in patients with NS, and the effect of the drug and the mechanism by which it improved the patient’s nephrotic condition in the present case are unknown. However, in our patient, the improvement in urinary protein level was observed concomitantly with a significant decrease in LDL after evolocumab administration. These findings strongly suggest that evolocumab contributed to the clinical improvement of the nephrotic condition.

NS with minimal change is a benign disease that immediately responds to steroids in many cases. However, in elderly patients and in intractable cases, we must address concerns about serious complications associated with treatment with steroids and immunosuppressive drugs. We believe that the findings presented here are unique and may be useful when treating similar cases, and we hope to review the findings of a few more similar cases in the future.

## Conclusion

We have described a case of refractory nephrotic syndrome successfully treated with a PCSK9 inhibitor. Aggressive cholesterol-lowering abilities of PCSK9 inhibitors may be beneficial in patients with refractory nephrotic syndrome, so findings from similar cases are anticipated in the future.

## Abbreviations

ACTH: Adrenocorticotropic hormone
CyA: Cyclosporin A
LDLA: Low-density lipoprotein apheresis
NS: Nephrotic syndrome
PCSK9: Proprotein convertase subtilisin/kexin type 9
PSL: Prednisolone
UP/UC: Urine protein/urine creatinine ratio
